# Pet ownership is associated with greater cognitive and brain health in a cross-sectional sample across the adult lifespan

**DOI:** 10.3389/fnagi.2022.953889

**Published:** 2022-10-20

**Authors:** Ian M. McDonough, Hillary B. Erwin, Nancy L. Sin, Rebecca S. Allen

**Affiliations:** ^1^Department of Psychology, The University of Alabama, Tuscaloosa, AL, United States; ^2^Alabama Research Institute on Aging, Tuscaloosa, AL, United States; ^3^Department of Psychology, University of British Columbia, Vancouver, BC, Canada

**Keywords:** brain age gap, independent component analysis, aging, protective factors (resilience), magnetic resonance imaging, resting state-fMRI

## Abstract

Human-animal interactions that stem from pet ownership have a wide range of benefits for social, emotional, and physical health. These factors also tend to improve cognition. Following this logic, owning a pet could indirectly enhance cognitive and brain health through mechanisms like improvements in well-being, socialization, and decreased stress. In the present study, cross-sectional data were drawn from the Alabama Brain Study on Risk for Dementia in which 95 participants aged 20–74 were recruited. Specifically, 56 adults were pet-owners and 39 adults were not pet-owners. Multivariate analyses revealed that pet ownership was related to higher levels of cognition and larger brain structures, and these effects were largest in dog owners. The most consistent cognitive relationships were found with better processing speed, attentional orienting, and episodic memory for stories, and with dorsal attention, limbic, and default mode networks. Moreover, we show that owning a pet can reduce one’s brain age by up to 15 years. Pet ownership was not related to indirect factors including social, emotional, and physical health. We found also that older adults’ brain health benefited from owning more than one pet versus owning one or fewer pets. These findings indicate that pet ownership, especially dog ownership, may play a role in enhancing cognitive performance across the adult lifespan, which could in turn influence protection against age-related cognitive decline.

## Introduction

According to the 2021–2022 National Pet Owners Survey, about 90.5 million families in the United States own a pet with nearly 70% of those being a dog (American Pet Products Association, 2021). Human-animal interactions that stem from pet ownership have a wide-range of benefits for social, emotional, and physical health (Hart, 1995; Wells, 2007; Perkins et al., 2008; Beetz et al., 2012; Beck, 2014; Ling et al., 2016). Pets enhance the social lives of older adults in many ways, including providing someone to talk to, presenting opportunities to meet new people on walks, and providing a topic of conversation (Beetz et al., 2012; Beck, 2014). All these factors can improve health by decreasing stress, increasing sleep quality, reducing blood pressure, and possibly improving one’s immune system functioning (Khan and Farrag, 2000; Headey and Grabka, 2006).

Behaviors that improve mental and physical functioning often improve cognition (Hertzog et al., 2008). Following this logic, owning a pet might indirectly confer protection against age-related declines in cognition through the aforementioned mechanisms. Researchers have also hypothesized that pet ownership might directly benefit cognition by enriching one’s environment and enhancing brain plasticity (Yorke, 2010). To take care of an animal, one needs to remember to feed, walk, and groom them. One must engage in critical thinking, plan for the future, and practice self-regulation when exerting patience with a pet (Ling et al., 2016). Because dogs tend to be more dependent than other pets like cats, some of these benefits may be more pronounced in dog owners than cat owners.

Despite these potential benefits of pet ownership, few studies have investigated the benefits of owning pets among community-dwelling older adults (Hughes et al., 2020; Albright et al., 2022). Most research investigating such relationships has focused on the therapeutic effects of animal-assisted therapy in patients with memory disorders (Kanamori et al., 2001; Bono et al., 2015) or those who need care in nursing homes (Stasi et al., 2004; Colombo et al., 2006). These studies investigated very coarse, global measures of cognition *via* measures like the Mini-Mental Status Exam (MMSE) and showed mixed results. Two notable studies that have investigated cognitive differences in community-dwelling older adults found small, but significant benefits for (1) executive function when comparing pet owners to non-pet owners (with no differences in dog versus cat owners; Branson et al., 2016) and (2) global cognition for those given an insect to care for compared to an older adult control group (Ko et al., 2016). This limited evidence for the benefits of pet ownership may be due to the focus on older adults after memory and health have already become sufficiently compromised or the use of insensitive, global tests of cognition.

**Figure 1:**
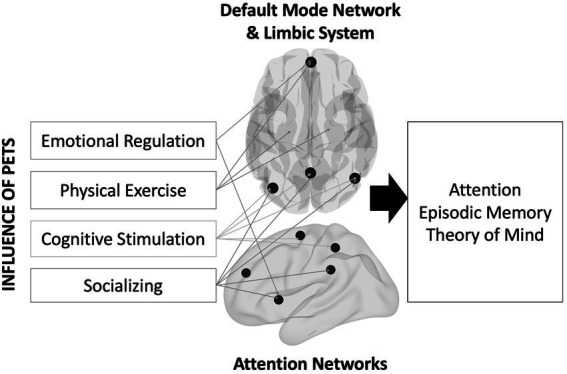
Theoretical model describing the influence of pet ownership on brain and cognitive health.

An unexplored reason for the lack of strong evidence might be a relative insensitivity of pet ownership on distal outcomes like cognition because such changes in cognition might take a long time to manifest (i.e., the effects might be latent). Instead, more proximal outcomes like brain structure and function might be more sensitive to the impacts of owning a pet. As shown in Figure 1, the social, emotional, and physical health benefits described earlier should impact multiple brain regions (Bickart et al., 2014; Puce et al., 2015; Porcelli et al., 2019). To socialize (with humans or pets), one must understand and think about the others’ perspective—known as Theory of Mind (Gallagher and Frith, 2003). Studies have shown that engaging in theory of mind activates overlapping brain regions involved with memory, namely the default mode network (DMN; Spreng et al., 2009). Regulating one’s emotions also activates parts of the DMN (e.g., ventromedial prefrontal cortex; Hostinar and Gunnar, 2015). Physical exercise interventions increase blood flow and oxygen delivery to the brain, thereby improving brain function in older adults (Bherer et al., 2013). Some of these regions include the ventromedial prefrontal cortex and the hippocampus. Lastly, older adults engaging in complex learning environments show increases in brain activity in posterior brain regions that overlap with the DMN, including lateral parietal cortex and the precuneus (McDonough et al., 2015).

Only a handful of studies have compared brain activity between pet and non-pet owners and many of them have focused on the neural correlates of visual perception (Mormann et al., 2011; Stoeckel et al., 2014). When viewing photographs of companion animals, pet-owners exhibited more brain activation than non-pet owners in lateral frontal and occipital regions, which are thought to be involved in attention and visual processing (Hayama et al., 2016). This study also showed that greater bonds with their pets were associated with higher brain activity in the lateral parietal cortex and the precuneus (regions in the DMN). These regions might be related to inferring mental states in pets. Supporting this idea, Desmet et al. (2017) showed that interpreting dog behaviors activated the DMN to a greater extent than interpreting human behaviors, especially when the behaviors were harder to interpret. These findings suggest that pets clearly attract the interest and attention of humans, but these studies do not reveal whether pet-ownership confers long-term alterations within brain networks when not actively viewing pets or other animals.

As a group, older adults show widespread brain shrinkage, cortical thinning, and reduction in within-network brain activity (Grady, 2012). However, some neurocognitive theories of aging hypothesize that middle-aged and older adults who can maintain optimal cognitive and brain health often show a “youth-like” pattern of brain structure and function (Park and Reuter-Lorenz, 2009; Nyberg et al., 2012). Pet ownership might serve as a series of experiences that enhances cognitive and brain functioning.

### Present study

First, we tested the extent that pet-ownership was associated with distal outcomes in specific cognitive domains (attention, episodic memory, and inductive reasoning). Additional proximal outcomes include brain structure (volume, surface area, and cortical thickness) and functional connectivity. Each neuroimaging modality was extracted from key cortical networks (DMN, limbic, and attention) to test targeted *a priori* hypotheses while reducing the number of multiple comparisons as would be done in a whole-brain approach. We hypothesized that owning a pet would be associated with (1) better cognitive functioning, (2) larger brain structures, and (3) higher within-network functional connectivity compared to not owning any pets. In sensitivity analyses, we also tested (a) whether the effects remained after removing non-dog owners from the analyses (i.e., directly comparing dog owners vs. non-pet owners), (b) whether dog-ownership differed from all others (both non-ownership and non-dog owners), and (c) whether the effects remained after controlling for global cognition/brain integrity.

Second, we tested a novel hypothesis that owning multiple pets might increase one’s daily cognitive load. Cognitive load might be elevated due to the different behaviors and needs of each pet. Accordingly, we predicted that owning more than one pet, regardless of type, will be associated with greater cognitive functioning and brain integrity than owning one or no pet. In sensitivity analyses, we tested whether the relationships remained after controlling for global cognition/brain integrity.

In exploratory analyses, we tested for interactions with age to examine whether the benefits of owning a pet differ depending on age group. Another way to investigate this question is to calculate a “brain age” score for each participant and test whether owning a pet leads to decelerated brain aging or accelerated brain aging. Brain age scores are created by comparing one’s predicted age based on brain measures and one’s chronological age as a summary score of brain health (Cole and Franke, 2017). Finally, we tested for evidence that any significant relationships can be accounted for by indirect effects of social, emotional, and physical health.

## Materials and methods

The hypotheses and planned analyses were pre-registered on the Open Science Framework at: https://osf.io/e7up3 to improve the integrity of research by preventing practices that allow for too many researcher degrees of freedom, thereby creating false positives (Simmons et al., 2021).

### Participants

Data were used from the Alabama Brain Study on Risk for Dementia (McDonough et al., 2019). One of the goals of the study was to uncover evidence for new potential protective factors for cognitive and brain health in middle-aged and older adults at varying risk for dementia along with a comparative sample of young adults. Participants were recruited from the Tuscaloosa and Birmingham areas through word of mouth, flyers, online ads, and newsletters. Participants were initially screened over the phone to assess study eligibility. Participants were included if they fell in the young adult age range (20–30) or were between 50 and 75 years old. Participants were excluded if they had contra-indicators for magnetic resonance imaging (MRI), were left-handed, had a prior diagnosis of any neurological condition, stroke, traumatic brain injury, claustrophobia, or history of substance abuse. Participants were then scheduled for a neuropsychological/physical health assessment and an MRI assessment. In between the two assessments, an online survey was given to assess sociodemographic information and various lifestyle factors including subjective well-being, social well-being, and psychological distress. After the neuropsychological assessment, participants were further excluded if they had a St. Louis University Mental Status (SLUMS; Tariq et al., 2006) score less than 20 (after adjusting for education level). All participants gave informed consent as approved by the institutional review board at The University of Alabama.

A power analysis using G*Power indicated that a total sample size of 78 participants would be needed to detect a large effect size (*d* = 0.80) with 80% power for an independent *t*-test (e.g., pet owner vs. non-owner) at an alpha level of 0.01. Ninety-five participants completed the neuropsychological assessment and 86 completed the MRI session. Participants’ vision was normal or corrected to normal using MR-compatible glasses or contact lenses. One participant was missing their years of education which was used as a covariate in all analyses. Regression-based imputation was used to estimate this value to maximize the sample size. The parameter estimates from this regression equation were then used to predict the participant’s education level.

### Cognitive assessments

Six cognitive domains were assessed: attention, processing speed, episodic memory, executive function, reasoning, and crystallized ability (Table 1). Composite scores were created when at least two tests represented a construct. For our primary hypotheses, we only tested whether pet ownership would be related specifically to attention/speed, episodic memory, and reasoning. The other cognitive domains were included in an analysis controlling for global cognition to investigate differential associations.

**Table 1:** Assessments.

**Assessments**	**Measure**	**Citation**
**Cognitive**		
Attention (ANT)		
Alerting	RT difference	Fan et al. (2002)
Orienting	RT difference	Fan et al. (2002)
Processing Speed		
Digit Comparison	Accuracy	Hedden et al. (2002) and Salthouse and Babcock (1991)
Trail Making Test A	RT	Reitan (1979)
Attention Network Test	Mean RT	Fan et al. (2002)
Episodic Memory (WMS-IV)		
Verbal (Paired Associates)	Accuracy	Drozdick et al. (2018)
Story	Accuracy	Drozdick et al. (2018)
Spatial	Accuracy	Drozdick et al. (2018)
Reasoning		
Raven’s Progressive Matrices	Accuracy	Raven (1938)
Crystallized Ability (WRAT-IV)		
Word Reading	Accuracy	Wilkinson and Robertson (2006)
Sentence Completion	Accuracy	Wilkinson and Robertson (2006)
Executive Function		
Trail Making Test B	RT	Reitan (1979)
Executive Attention (ANT)	RT difference	Fan et al. (2002)
**Physical Health**		
Body Mass Index	OMRON Scale	–
Blood Pressure	HealthSmart Monitor	–
Physical Activity Form	Mean	Taylor et al. (2005)
**Psychosocial**		
Subjective Well-being		
Positive and Negative Affect Schedule	Mean	Watson et al. (1988)
Satisfaction with Life Scale	Mean	Diener et al. (1985)
Social Well-being		
UCLA Loneliness Scale (Revised)	Mean	Russell (1996)
Interpersonal Support Evaluation List	Mean	Cohen and Hoberman (1983)
Psychological Distress		
Center for Epidemiological Studies–Depression	Sum	Radloff (1977)
Perceived Stress Scale (short)	Sum	Cohen et al. (1983) and Warttig et al. (2013)
Short-Form Health Survey	Sum	Ware and Sherbourne (1992)

ANT, Attention Network Test; WMS, Wechsler Memory Scale IV Edition; WRAT, Wide Range Achievement Test IV Edition; RT, Reaction time.

Three different types of attention were assessed using the Attention Network Test (ANT; Fan et al., 2002): alerting, orienting, and executive attention. Response times derived from difference scores were used as outcome variables. Processing speed was assessed using the Digit Comparison Task (Salthouse and Babcock, 1991; Hedden et al., 2002), Trail Making Test A (Reitan, 1979), and mean response times from the Attention Network Test (Fan et al., 2002). Response times were square root transformed and reversed. Episodic memory was assessed using the Wechsler Memory Scale IV (Drozdick et al., 2018) and tested verbal, story, and spatial memory. Each of the three memory tests consisted of an immediate recall test, a delayed recall test, and a delayed recognition test, which were standardized and averaged to create composite scores. Preliminary correlations between each test were low, suggesting that separate composite scores would be most appropriate. Reasoning was assessed using Raven’s Progressive Matrices (Raven, 1938). Accuracy was used as the primary outcome variable. The additional cognitive tasks were used to form a global cognition score. These included a word reading and sentence completion task from the Wide Range Achievement Test-Fourth Edition (WRAT-4; Wilkinson and Robertson, 2006) to form a crystallized ability score. Executive function was measured using the Trail Making Test B and the executive attention score from the ANT.

### Physical health assessments

Several health assessments were administered as a break in between the cognitive sessions (Table 1). Height was self-reported by participants. Weight was assessed using an OMRON body composition BF511 scale (Omron, Japan). Body mass index (BMI) was calculated as weight (kg)/height (m)^2^. Waist circumference was assessed by trained study staff who measured circumference at the level of the navel. A HealthSmart semi-automatic blood pressure monitor was used to estimate systolic and diastolic blood pressure after being seated for at least 20 min. When possible, two measures were taken at the separate assessment sessions and averaged together. Lastly, in an online survey, subjects were asked several questions that were used in the Jackson Heart Study (Taylor et al., 2005) regarding their health habits. The Physical Activity Form was used to measure the frequency that participants engage in physical activity during their leisure time; each item used a five-point scale: less than once a month, once a month, 2–3 times a month, once a week, and more than once a week.

### Psychosocial assessments

Several questionnaires were administered in an online survey between the cognitive and MRI sessions. Several were chosen to represent *a priori* constructs related to subjective well-being, social well-being, and psychological distress (Table 1). Pet ownership has been previously associated with higher well-being and reduced psychological distress. For each construct, each scale was standardized, reversed if appropriate, and averaged together. Two questionnaires were used to assess subjective well-being: the Positive and Negative Affect Schedule (PANAS; Watson et al., 1988) and the Satisfaction with Life Scale (SWLS; Diener et al., 1985). Higher scores indicated greater subjective well-being. Two questionnaires were used to assess social well-being: the Revised UCLA Loneliness Scale (Russell, 1996) and a short form of the Interpersonal Support Evaluation List (ISEL; Cohen and Hoberman, 1983; Payne et al., 2012). Higher scores indicated greater social well-being. Three questionnaires were used to assess psychological distress: Center for Epidemiological Studies–Depression (CES-D) Scale (Radloff, 1977), the short-Perceived Stress Scale (PSS-4; Cohen et al., 1983; Warttig et al., 2013), and three questions assessing feelings of anxiety or depression from the Short-Form Health Survey (SF-36; Ware and Sherbourne, 1992). Higher scores represented greater distress.

### MRI assessments

Participants underwent one structural MRI, task-related fMRI (not reported here) and two 5-min resting state scans. The present analysis focused on structural and resting-state scans.

#### Structural MRI acquisition and processing

MRI data were collected using a 3T Siemens PRISMA scanner at the UAB Civitan International Neuroimaging Laboratory. High resolution T1-weighted structural MPRAGE scans were acquired using (parallel acquisition acceleration type = GRAPPA; acceleration factor = 3, TR = 5,000 ms, TE = 2.93 ms, TI 1 = 700 ms, TI 2 = 2,030 ms, flip angle 1 = 4°, flip angle 2 = 5°, FOV = 256 mm, matrix = 240 × 256 mm^2^, in-plane resolution = 1.0 × 1.0 mm^2^).

T1 structural images were visually inspected for signs of movement or artifact. Structural images were then preprocessed in FreeSurfer version 6.0 (Fischl, 2012). The Freesurfer recon-all pipeline transformed the raw imaging data with a series of processes, including affine registration onto Talairach atlas, bias correction, skull stripping, and removing brain stem and cerebellum. Mindcontrol (Keshavan et al., 2018) was used to detect outlying regions and manual corrections were implemented to correct for any errors. Volumes transformed to the default surface-based map and network templates from Yeo et al. (2011) were used to extract mean gray matter volume, surface area, and cortical thickness values for each network. Intracranial volume was regressed out of all gray matter volume and area estimates as recommended by Buckner et al. (2004) before entering them into our main analyses.

#### Functional MRI acquisition and processing

Functional scans used T2*-weighted EPI sequences (56 interleaved axial slices, 2.5 mm thickness, TR = 1,720 ms, TE = 35.8 ms, flip angle = 73°, FOV = 260 mm, matrix = 104 × 104 mm, in-plane resolution = 2.5 × 2.5 mm^2^, multi-band acceleration factor = 4). Functional data were unwarped, realigned, and spatially smoothed using an 8 mm FWHM kernel using the CONN toolbox (Whitfield-Gabrieli and Nieto-Castanon, 2012). These smoothed images were then denoised using Multivariate Exploratory Linear Optimized Decomposition into Independent Components (MELODIC). Specifically, in-house scripts were used to flag spatiotemporal components that applied machine learning to frequency and temporal elements indicative of potential artifacts. The flagged components were then regressed from the BOLD signal using MELODIC. The denoised data were warped into a study template using Advanced Normalization Tools (ANTS; Avants et al., 2009). The CONN Toolbox was used to regress out white matter and CSF BOLD time series, motion parameters and their first derivatives, and outlying time points and their first derivatives (CONN’s version of scrubbing). Bandpass filtering was implemented with a lowpass of 0.008 Hz and a highpass of 0.09 Hz. Quadratic detrending and despiking were also implemented. Finally, the first scan was removed from all sessions and the two scans were concatenated before analysis.

Within-network functional connectivity was computed using the Dual_Regression function in FSL to extract the time series and spatial correlation maps that best fit a volume-based atlas of the 17 networks from Yeo et al. (2011). The dual regression resulted in Z-scored subject-specific maps for each network. To summarize connectivity, the average Z-score was computed for each subject’s map using each network template as a mask. Higher scores represent stronger within-network connectivity. Although we calculated within-network connectivity as defined by the original templates, many of these networks also were highly correlated with subcortical regions. The average connectivity values in subcortical brain region for each network tested can be found in Supplementary material.

### Statistical analysis

Cognition and brain health were assessed using multivariate analyses of covariate (MANCOVAs) separately for each modality (cognition, brain volume, surface area, cortical thickness, and resting-state functional connectivity). The first family of tests investigated the effects of pet ownership (the independent variable) while controlling for *a priori* variables chronological age, years of education, ethnoracial group (1 = non-Hispanic White, 0 = others), and sex (1 = Male, 0 = Female), implementing Bonferroni corrections for five comparisons. If the multivariate statistic was significant, follow-up analyses of covariance (ANCOVAs) were conducted for each individual measure.

These analyses were followed by sensitivity analyses to test whether similar effects would be found for (a) dog owners compared with non-dog owners (pet owners and non-pet owners), (b) dog owners with non-pet owners, and (c) pet ownership with non-pet ownership after controlling for a global measure of the current metric (cognition, brain volume, etc.). Global scores were calculated by standardizing all cognitive measures or brain networks and averaging them together. These global scores were regressed out of each measure and the MANCOVA was recalculated. Given the goal of the sensitivity analyses were to understand general trends of variations in analyses, we report uncorrected *p*-values for the MANOVA and only correct for multiple comparisons in follow-up ANOVAs.

The second family of tests used a series of MANCOVAs to investigate whether owning more than one pet was associated with better cognitive or brain health compared with owning one or no pets, implementing Bonferroni corrections for five comparisons. The only sensitivity analysis that was appropriate was investigating the effects of owning multiple pets while also controlling for global cognitive or brain health.

Lastly, exploratory analyses were conducted to test whether chronological age moderated any of the effects, whether owning a pet was associated with a younger “brain age,” and whether previously found health and psychosocial variables indirectly related pet ownership to cognitive or brain health. Even though these analyses were exploratory, they were all planned exploratory analyses except for the BrainAGE analysis (for review, see Franke and Gaser, 2019). For this analysis, we created a matrix of all cognitive and brain network scores and regressed out the covariates for participants that had each score (*N* = 83). We then centered and scaled this matrix and used linear support vector regression with default parameters to assess a brain age score for each participant. A leave-one-participant-out method was used such that all the available data was used to create an age model. The left-out participant then served as the test item to apply that model in an unbiased way and to determine a predicted brain age score. This process was applied repeatedly leaving out a new participant each time, resulting in 83 models. A brain age correction factor was then implemented (as discussed in de Lange and Cole, 2020) to correct for extreme estimations that often occur at the lowest and highest ends of the age continuum. The gap between the predicted age and chronological age represents an accelerated or decelerated biological age (i.e., BrainAGE) relative to one’s actual age.

To test for indirect effects, four *a priori* indirect constructs were assessed: physical health, subjective well-being, social well-being, and psychological distress. All analyses were carried out in R (R Core Team, 2021).

## Results

### Sociodemographic descriptive statistics

Compared with non-pet owners, pet owners rated themselves higher on perceived social class (*t* (76.34) = 2.50, *p* = 0.014) and were more likely to be non-Hispanic White (*χ*^2^(1) = 6.05, *p* = 0.014). Compared with owning one or fewer pets, owning multiple pets was more likely for females than males (*χ*^2^(1) = 5.51, *p* = 0.019) and for non-Hispanic White Americans than ethnoracial minorities (*χ*^2^(1) = 9.77, *p* = 0.0018). No other variables were significant (*p*s > 0.065; Table 2).^1^ Mean values of brain metrics can be found in Supplementary material.

**Table 2:** Sociodemographic descriptives.

	Pet ownership categories					
	Non-pet owner	Pet owner	Non-dog owner	Dog-owner	Own 1 or no pets	Own more than 1 pets
	*M* (SD), *N*, %	*M* (SD), *N*, %	*M* (SD), *N*, %	*M* (SD), *N*, %	*M* (SD), *N*, %	*M* (SD), *N*, %
*N* total	39	56	52	43	65	30
*N* MRI scans	35	51	46	40	58	28
Number of pets	0.00 (0.00)	2.11 (0.18)	0.50 (0.16)	2.14 (0.20)	0.4 (0.06)	3.07 (0.22)
Age	54.62 (2.7)	48.95 (0.35)	54.27 (2.29)	47.65 (0.75)	50.26 (2.27)	53.47 (0.83)
Education (years)	14.90 (0.44)	14.25 (0.33)	14.4 (0.39)	14.65 (0.34)	14.57 (0.34)	14.40 (0.39)
Non-Hispanic White (%)	0.46	0.73	0.48	0.79	0.51	0.87
Sex (% Female)	0.49	0.70	0.54	0.70	0.52	0.80
Body mass index	27.69 (0.94)	27.63 (0.84)	27.97 (0.89)	27.27 (0.87)	27.75 (0.80)	27.45 (0.96)
Abdominal circumference (cm)	103.76 (2.46)	97.65 (0.68)	103.48 (2.09)	96.14 (0.25)	102.13 (2.01)	95.88 (0.06)
SES ladder (1–10)	3.87 (0.39)	5.11 (0.30)	4.06 (0.32)	5.26 (0.35)	4.32 (0.29)	5.20 (0.45)
Salary level	2.78 (0.35)	2.88 (0.28)	2.71 (0.29)	3.00 (0.33)	2.63 (0.26)	3.27 (0.38)
Household income level	3.31 (0.35)	4.06 (0.33)	3.26 (0.30)	4.32 (0.38)	3.43 (0.29)	4.38 (0.43)
Financial strain	6.46 (0.54)	5.73 (0.42)	6.60 (0.49)	5.35 (0.42)	6.34 (0.41)	5.37 (0.57)
Wears fitbit-type device	1.18 (0.06)	1.16 (0.05)	1.19 (0.06)	1.14 (0.05)	1.17 (0.05)	1.17 (0.07)

Salary level and household income level ranged from 1 (<$15,000/year) to 8 (>$150,000/year); Financial strain ranged from 4 (lowest difficulty paying bills) to 16 (highest difficulty paying bills).

### Effects of pet ownership on cognitive and brain health

#### Cognitive health

**Figure 2:**
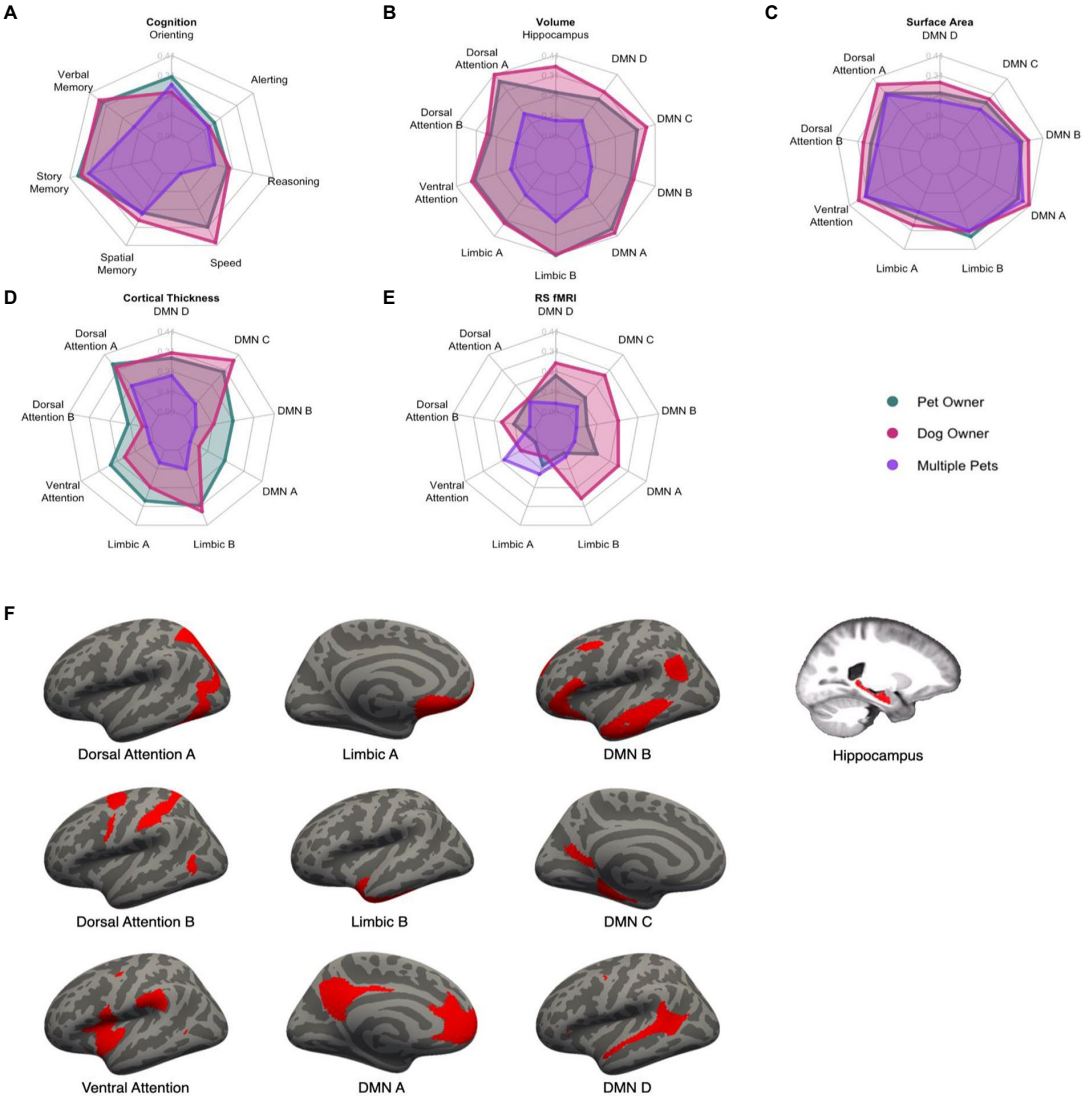
Radar plots of the standardized effect sizes of pet ownership (green), dog ownership (red), and owning multiple pets (purple) for cognitive and brain outcomes **(A–E)**. Higher values indicate a greater difference between the three groups and non-pet owners. Visual depiction of the networks used in the analyses **(F)**. DMN, Default Mode Network.

A MANCOVA was conducted to investigate the effect of pet ownership on seven measures of cognition, controlling for chronological age, years of education, racial category, and biological sex (Table 3). This analysis resulted in a significant multivariate effect of pet ownership, Pillai’s Trace = 0.27, *F*(7, 81) = 4.25, *p_adj_* = 0.0024. Follow-up univariate analyses showed that pet ownership was associated with greater attentional orienting (*β* = 0.30, 95% CI [0.10, 0.47], *p_adj_* = 0.029), processing speed (*β* = 0.31, 95% CI [0.11, 0.47], *p_adj_* = 0.024), verbal memory (*β* = 0.31, 95% CI [0.11, 0.48], *p_adj_* = 0.0098), and story memory (*β* = 0.34, 95% CI [0.15, 0.50], *p_adj_* = 0.0026). Details can be found in Figure 2 (Panel A).

**Table 3:** Summary of multivariate statistics by group membership.

Outcome category	Pet ownership^a^			Dog ownership^a^	Multiple pet ownership^b^		
	ME	CG	INT	ME	ME	CG	INT
Cognitive health	•	•		•	•	•	
Brain volume	•	•		•			
Surface area		•		•			
Cortical thickness				•			•
RS connectivity							

a Reference category was non-pet owners;

b Reference category was one or no pet owners; RS, Resting-state; ME, Main effect; CG, Main effect controlling for global cognition or brain integrity; INT, Interaction with age; •, Significant effect.

#### Brain health

For brain volume, the MANCOVA resulted in a significant effect of pet ownership, Pillai’s Trace = 0.32, *F*(10, 71) = 3.30, *p_adj_* = 0.0075. Pet ownership was associated with larger volumes in the dorsal attention A (*β* = 0.37, 95% CI [0.16, 0.53], *p_adj_* = 0.0068), ventral attention (*β* = 0.32, 95% CI [0.11, 0.49], *p_adj_* = 0.035), limbic A (*β* = 0.32, 95% CI [0.11, 0.49], *p_adj_* = 0.035), limbic B (*β* = 0.41, 95% CI [0.22, 0.56], *p_adj_* = 0.0012), default mode A (*β* = 0.37, 95% CI [0.16, 0.53], *p_adj_* = 0.0072), and default mode C (*β* = 0.32, 95% CI [0.11, 0.49], *p_adj_* = 0.035) networks. The MANCOVA did not reach the criterion for significance for pet ownership on surface area (Pillai’s Trace = 0.24, *F*(9, 72) = 2.51, *p_adj_* = 0.075), cortical thickness (Pillai’s Trace = 0.23, *F*(9, 72) = 2.37, *p_adj_* = 0.11), or resting-state connectivity (Pillai’s Trace = 0.086, *F*(9, 72) = 0.76, *p_adj_* = 1.00). Standardized effect sizes for each of the brain measures are shown in Figure 2.

### Sensitivity analyses for effects of pet ownership

We conducted three additional planned analyses to assess the robustness of the effects by testing (a) dog-owners vs. non-dog owners (but could include other pets), (b) dog-owners vs. non-pet owners, and (c) pet-owners vs. non-pet owners after controlling for global cognition and brain integrity.

#### Cognitive health

The first compared dog owners with non-dog owners and yielded a significant effect of dog ownership (Pillai’s Trace = 0.28, *F*(7, 81) = 4.56, *p_uncorr_* = 0.00025). In follow-up ANOVAs by measure, processing speed, verbal memory, and story memory reached significance (*ps_adj_* < 0.005; Figure 2). The second analysis compared dog owners with non-pet owners and yielded a significant effect of dog owners (Pillai’s Trace = 0.35, *F*(7, 69) = 5.20, *p_uncorr_* = 0.000086). Attentional orienting and story memory reached significance (*ps_adj_* < 0.037). The last analysis intended to test whether pet ownership would be significant after controlling for global cognition. This analysis yielded a significant effect of pet ownership (Pillai’s Trace = 0.20, *F*(7, 81) = 2.86, *p_uncorr_* = 0.010). Only attentional orienting (*p_adj_* = 0.0083) and story memory (*p_adj_* = 0.037) were significant. These analyses suggest that the effects of pet ownership on cognition are quite robust and even stronger for dog owners than other pet owners. Moreover, pet ownership has differentially strong effects on story memory even after accounting for global cognition.

#### Brain health

For brain volume, dog owners showed a significant multivariate effect compared with non-dog owners (Pillai’s Trace = 0.33, *F*(10, 71) = 4.22, *p_uncorr_* = 0.00079). The same networks were significant as in the primary pet owner analysis (*ps_adj_* < 0.026) and bilateral hippocampal volume also was significant (*β* = 0.35, 95% CI [0.15, 0.52], *p_adj_* = 0.011). Dog owners also showed a significant multivariate effect compared with non-pet owners (Pillai’s Trace = 0.33, *F*(10, 71) = 4.22, *p_uncorr_* = 0.00079) with the same significant networks (*ps_adj_* < 0.017) but not the hippocampus (*p_adj_* = 0.060). After controlling for global brain volume, pet ownership continued to be significant (Pillai’s Trace = 0.27, *F*(10, 71) = 2.63, *p_uncorr_* = 0.0088) for the dorsal attention A, limbic B, and default mode A networks (*ps_adj_* < 0.022).

For surface area, dog owners showed a significant multivariate effect compared with non-dog owners (Pillai’s Trace = 0.28, *F*(9, 72) = 3.16, *p_uncorr_* = 0.0029) with significant effects in the dorsal attention A, ventral attention, limbic B, default mode A, and default mode B networks (*ps_adj_* < 0.037). Dog owners also showed a significant multivariate effect compared with non-pet owners (Pillai’s Trace = 0.32, *F*(9, 61) = 3.14, *p_uncorr_* = 0.0036) for the same significant networks (*ps_adj_* < 0.022). After controlling for global surface area, pet ownership was now significant (Pillai’s Trace = 0.25, *F*(9, 72) = 2.68, *p* = 0.0096) for the same significant networks as for dog-owners (*ps_adj_* < 0.048).

For cortical thickness, dog owners showed a significant multivariate effect compared with non-dog owners (Pillai’s Trace = 0.33, *F*(9, 72) = 3.79, *p_uncorr_* = 0.00038) with significant effects in the dorsal attention A, limbic B, and default mode C networks (*ps_adj_* < 0.024). Dog owners also showed a significant multivariate effect compared with non-pet owners (Pillai’s Trace = 0.33, *F*(9, 61) = 3.31, *p_uncorr_* = 0.0024) for the same significant networks (*ps_adj_* < 0.016). After controlling for global cortical thickness, pet ownership was not significant (Pillai’s Trace = 0.18, *F*(9, 72) = 1.62, *p_uncorr_* = 0.13). For resting-state connectivity, no multivariate effects were found for the subsequent sensitivity analyses (*ps*_uncorr_ > 0.22).

### Effects of owning multiple pets on cognitive and brain health

#### Cognitive health

The same MANCOVA was conducted as in the previous analyses. This resulted in a significant multivariate effect of owning multiple pets compared with owning one or no pets, Pillai’s Trace = 0.23, *F*(7, 81) = 3.52, *p_adj_* = 0.012 (Figure 2). Multiple pet ownership was associated with better story memory (*β* = 0.32, 95% CI [0.11, 0.48], *p_adj_* = 0.019).

#### Brain health

No multivariate effect of owning multiple pets was found on brain volume compared with owning one or no pets (Pillai’s Trace = 0.17, *F*(10, 71) = 1.42, *p_adj_* = 0.95), surface area (Pillai’s Trace = 0.13, *F*(9, 72) = 1.15, *p_adj_* = 1.00), cortical thickness (Pillai’s Trace = 0.13, *F*(9, 72) = 1.18, *p_adj_ = 1.00*), nor resting-state connectivity (Pillai’s Trace = 0.11, *F*(9, 72) = 1.02, *p_adj_* = 1.00).

### Sensitivity analyses for effects of owning multiple pets

#### Cognitive health

After controlling for global cognition, this analysis yielded a significant effect of owning multiple pets (Pillai’s Trace = 0.24, *F*(7, 81) = 3.71, *p_adj_* = 0.008). In follow-up ANOVAs, story memory and spatial memory were significant (*ps_adj_* < 0.034).

#### Brain health

After controlling for the relevant global brain integrity score, no multivariate effect of owning multiple pets was found on brain volume (Pillai’s Trace = 0.18, *F*(10, 71) = 1.50, *p_adj_* = 0.80), surface area (Pillai’s Trace = 0.15, *F*(9, 72) = 1.35, *p_adj_* = 1.00), cortical thickness (Pillai’s Trace = 0.14, *F*(9, 72) = 1.34, *p_adj_* = 1.00), nor resting-state connectivity (Pillai’s Trace = 0.11, *F*(9, 72) = 0.98, *p_adj_* = 1.00).

### Exploratory analyses

#### Interactions with age

**Figure 3:**
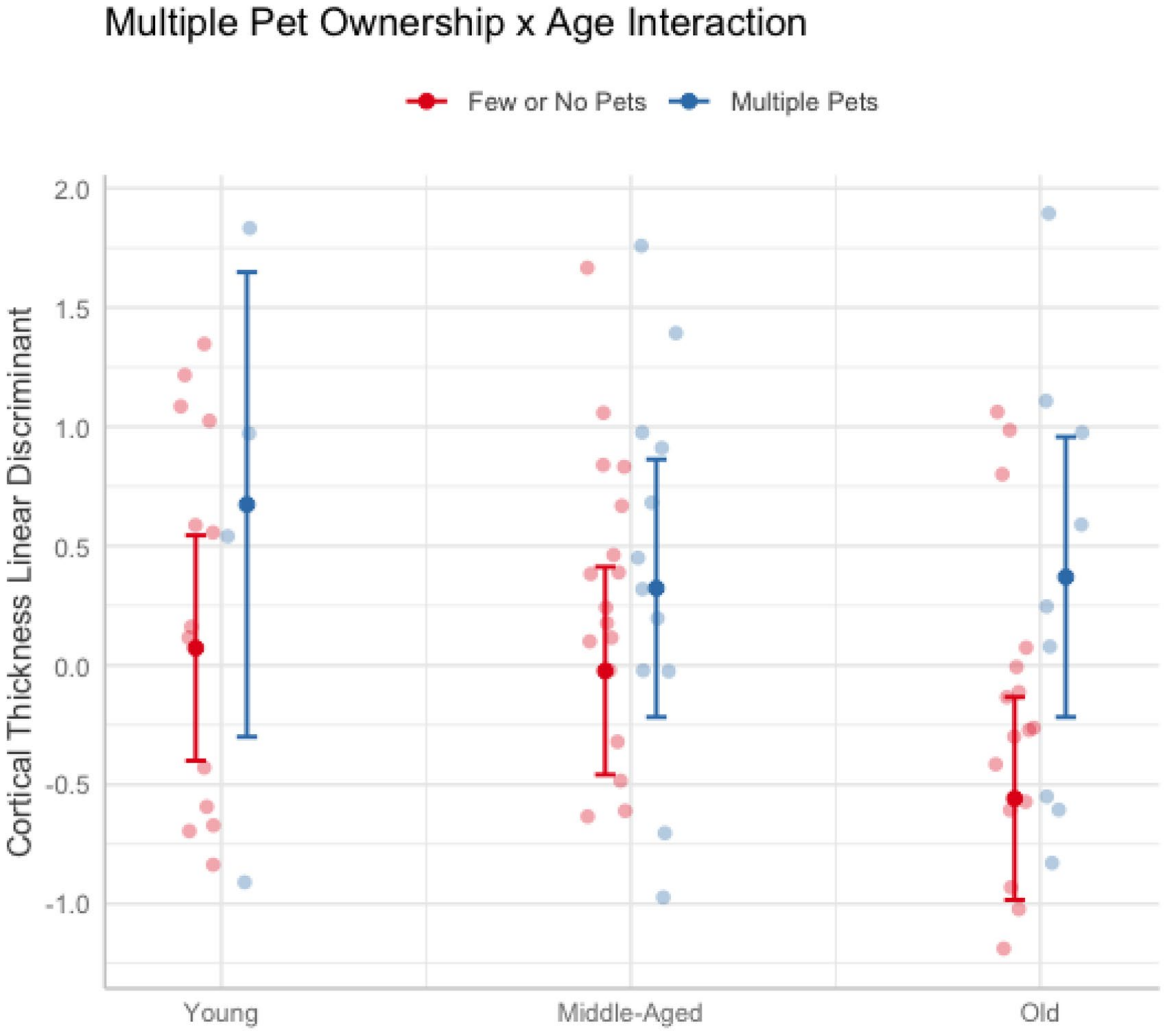
Plot illustrating the significant interaction between owning multiple pets and age on cortical thickness. Older adults (62 and older) who owned more than one pet (blue) had greater cortical thickness than older adults who owned one or no pets (red). The effect of multiple pet ownership was smaller in younger (aged 20–30) and middle-aged adults (aged 50–61). Error bars represent 95% confidence intervals.

In these analyses, age was treated as a continuous variable. Across cognitive and brain health measures, only cortical thickness revealed interactions with age when owning multiple pets (Pillai’s Trace = 0.23, *F*(9, 71) = 2.29, *p_uncorr_* = 0.026). Follow-up univariate tests were not significant (*ps_uncorr_* > 0.17). Thus, to better understand the nature of the results, a linear discriminant analysis was conducted on the cortical thickness measures after regressing out education, race, and sex. The largest coefficients were the dorsal attention A (12.90), default mode D (8.50), and limbic A (4.95) networks. Figure 3 breaks down age into categorial groups to show the nature of the interaction. The general trend was that owning multiple pets was associated with greater cortical thickness more so in older adults than middle-aged or young adults (Figure 3). No other interactions with age were found for pet ownership (*p*s > 0.10), dog ownership (*p*s > 0.15), or owning multiple pets (*p*s > 0.39).

#### BrainAGE gap

**Figure 4:**
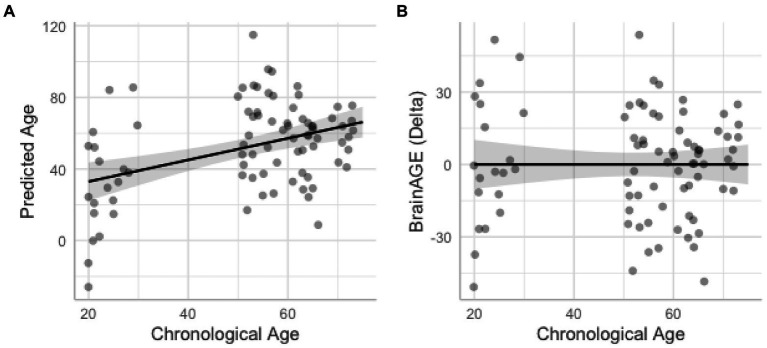
Scatterplots show correlations between chronological age and predicted age from the support vector machine model **(A)** and BrainAGE with higher difference scores representing accelerated brain aging **(B)**. Shaded areas represent 95% confidence intervals.

**Figure 5:**
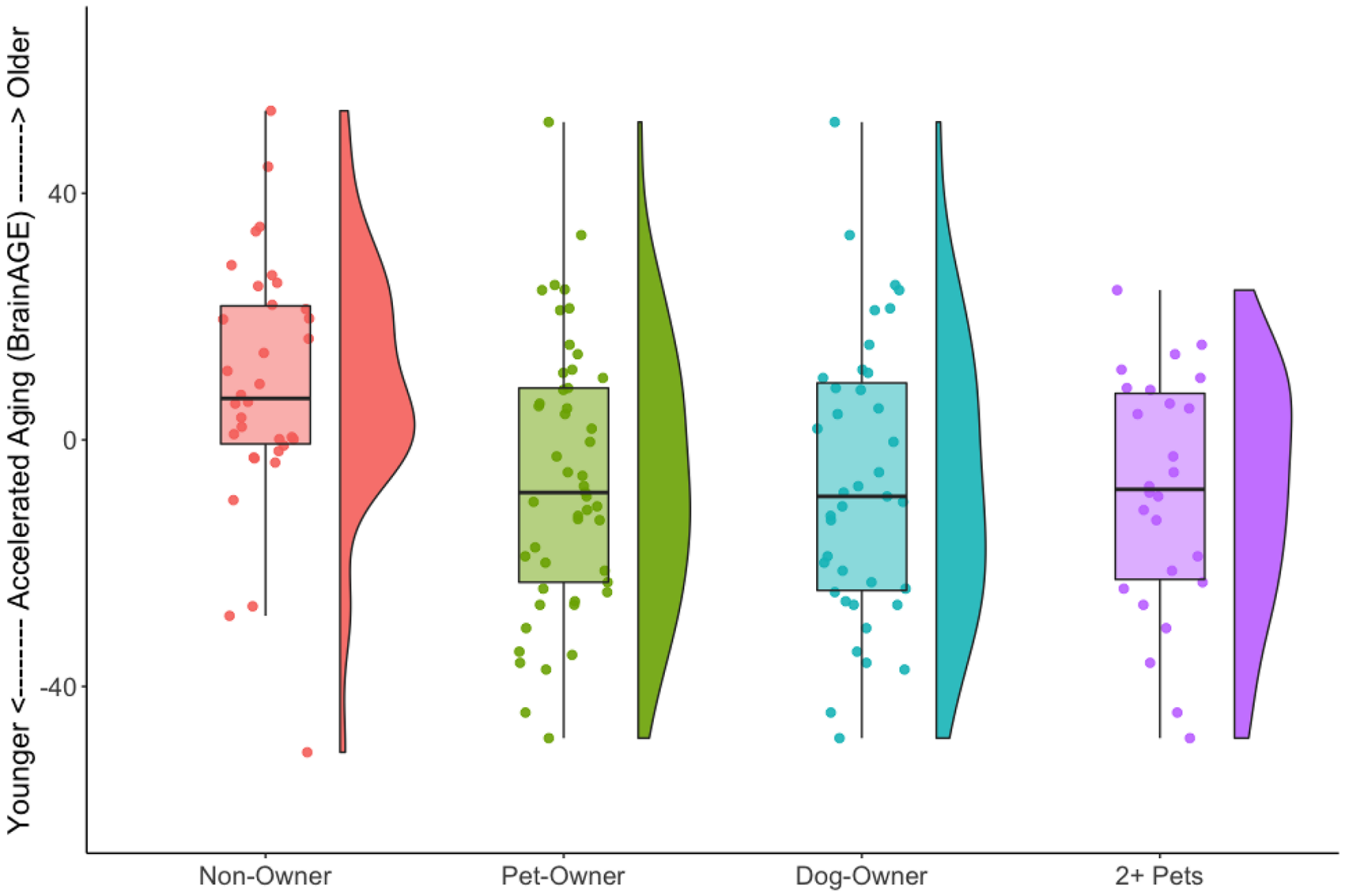
Raincloud plots illustrating BrainAGE scores as a function of pet ownership group. For each group, the left side shows a box plot and jittered instances of each participant and the right side shows a density plot. Non-pet owners had a higher BrainAGE score than the various pet owner groups.

**Figure 6:**
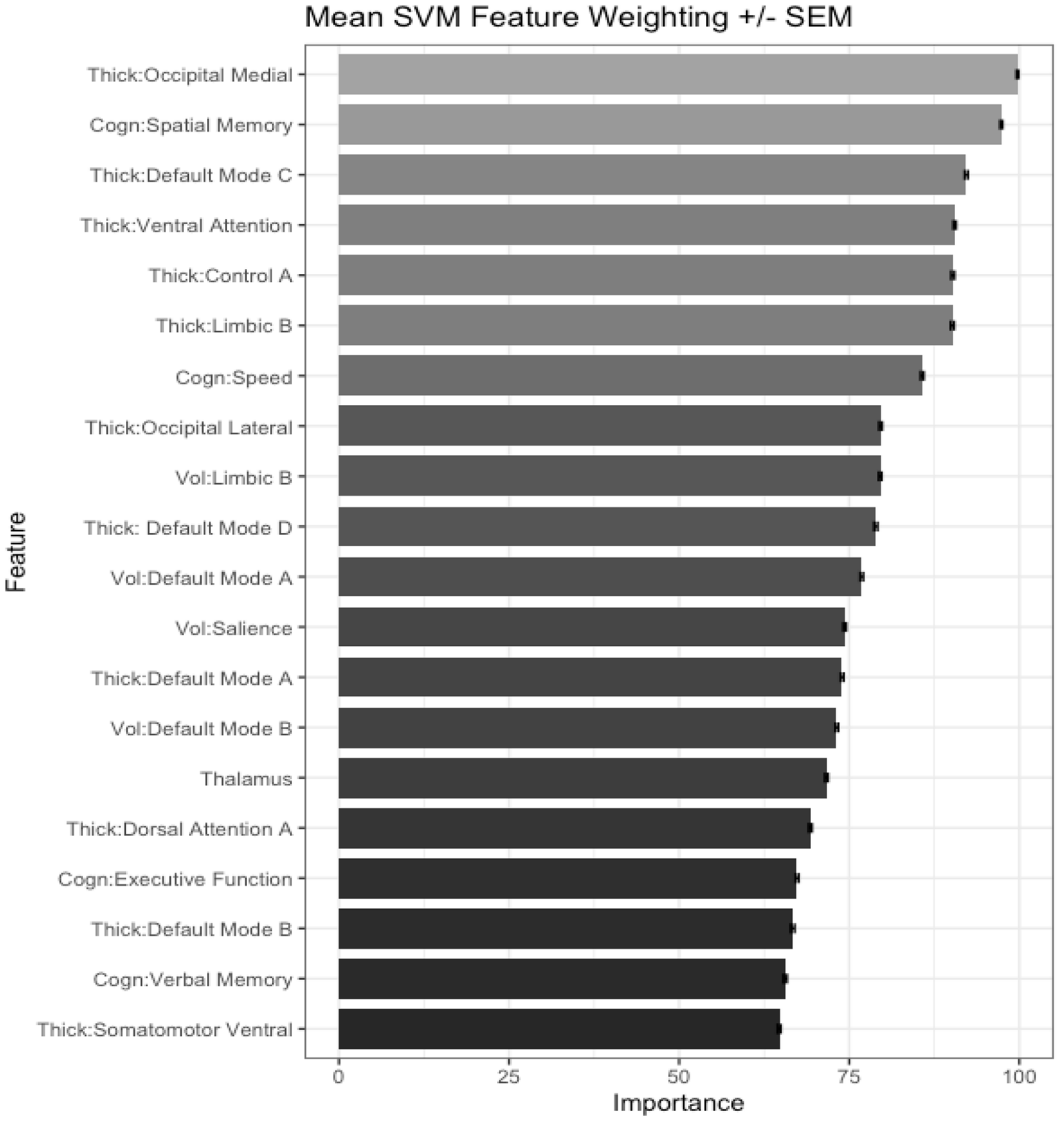
Bar plots showing the top 20 important features in the BrainAGE algorithm.

We also tested the extent that owning a pet was associated with a younger “brain age.” Chronological age was correlated with predicted age, *r* (81) = 0.41, *p* = 0.00015, 95% CI [0.21, 0.57] (Figure 4A). As in prior work (Smith et al., 2019), the BrainAGE score (i.e., the corrected difference between chronological age and predicted age) was not correlated with chronological age, *r* (81) = 0.00, *p* = 1.00 (Figure 4B). Independent *t*-tests were conducted to test for differential brain aging as a function of pet ownership. As can be seen in Figure 5, pet owners had a lower BrainAGE score than non-pet owners (*Mean Difference* = 15.10 years, *t* (73.28) = 3.22, *p_uncorr_* = 0.0019, *p_adj_* = 0058), dog owners had a lower BrainAGE score than non-pet owners (*Mean Difference* = 12.95 years, *t* (76.76) = 2.72, *p_uncorr_* = 0.0082, *p_adj_* = 0.025), and multiple pet owners than participants with one pet or no pets (*Mean Difference* = 12.56 years, *t* (56.69) = 2.60, *p_uncorr_* = 0.012, *p_adj_* = 0.035). The top 20 features that contributed to the BrainAGE score can be found in Figure 6. More than half of the top 20 consisted of cortical thickness measures in occipital, default mode, and attention networks. Thus, consistent with the notion of pet ownership as a protective factor of biological aging, owning a pet reduces one’s brain age up to 15 years, according to this metric.^2^

#### Indirect effects of pet ownership relationships on cognitive and brain health

We next investigated indirect effects through physical health, subjective well-being, social well-being, and psychological distress. None were related to pet ownership (*ps* > 0.31), dog ownership (*ps* > 0.32), or owning multiple pets (*ps* > 0.06). Thus, we did not conduct further analyses.

## Discussion

This study is the first to investigate a comprehensive analysis of the associations between pet ownership and both cognitive and brain health. In overarching multivariate analyses, we found clear support for the association between pet ownership and cognitive/brain health in an adult sample ranging up to 74 years. These effects were strongest for dog owners, followed by all pet-owners, and some effects were found for those owning multiple pets. Establishing these strong links are critical to inform neurocognitive interventions and to better understand potential brain mechanisms underlying the benefits of pet ownership.

### Specific cognitive relationships with pet ownership

Although many of the cognitive domains exhibited moderate effect sizes, the most consistent relationships were found with better processing speed, attentional orienting, and episodic memory for stories. Processing speed was measured through multiple reaction time tests. In the context of pet ownership, one might need to react quickly to alter a pet’s behavior (e.g., preventing ingestion of poisonous foods or clawing at furniture). We note that these processing speed tasks tap into both cognitive and motor speed–both of which may be needed when interacting with pets.

Attentional orienting occurs when a cue in one’s environment helps guide where attention should be directed next. In the case of pet ownership, owners often need to read the cues of their pets or the environment to predict how their pet might behave and adjust their actions accordingly (e.g., tightening their grip on a leash upon seeing a nearby cat). Stories often include an ordered and contextualized sequence of events. Memory for such stories might be needed for a variety of purposes in the context of pet ownership. When communicating to others about their pets, people often do so through storytelling of interesting (or comical) events, thus requiring remembering the original event in story form. When communicating to veterinarians, stories about recent unusual behaviors and context might help to better understand the underlying medical issue. Remembering previous sequences of events with one’s pet also aids in putting ourselves in their shoes (i.e., theory of mind) to consider their point of view and predict their future actions in similar situations. For these cognitive domains, repeated and sustained use might lead to long term benefits. Moreover, attentional orienting and story memory continued to be significant even after controlling for global cognitive performance, suggesting a differentially strong and specific effect for these domains.

### Specific brain relationships with pet ownership

The brain associations with pet ownership strongly align with the cognitive associations. The most robust brain networks that were associated with larger brain structures include the dorsal attention A (largely the posterior parietal), limbic B (temporal), and default mode A (medial prefrontal and parietal) networks. Whereas pet ownership showed moderately strong relationships only with brain volume, the same networks also were found for surface area and cortical thickness in the analysis comparing dog owners to non-dog owners and non-pet owners.

Collectively, these networks resemble two core brain systems involved in attention and episodic memory: the anterior temporal (AT) system and the posterior medial (PM) system (Ranganath and Ritchey, 2012). Specifically, the anterior portion of the default mode A network and the lateral temporal lobe from the limbic B network make up the AT system. This system is purportedly involved in processing familiarity signals of memory, emotional processing, and social cognition to aid in making inferences about the relevance or intentions of another person, animal, or other object (Ranganath and Ritchey, 2012). In contrast, the PM system has been implicated in retrieving past recollections and simulating possible future events (Ritchey and Cooper, 2020). Both processes involve creating a type of situation model that binds temporal, spatial, and sensory details (like in a story). Thus, the PM system is thought to create such mental situation models and apply those models to the current situation. Through functional interactions with the AT system, a person can create situation models of their own potential actions and those of others. Pet owners, especially dog owners, often attempt to read signals from their pets, create a mental model through anthropomorphism, and predict pets’ current and future needs.

### Effects of owning more than one pet

The present study proposed a novel hypothesis that owning more than one pet may confer additional benefits than owning one pet. Specifically, we proposed that owning more than one pet requires additional cognitive resources to juggle the responsibilities and understand the unique needs of each pet. Although we found a significant multivariate effect on cognition, the effect sizes were much smaller and only story memory survived multiple comparisons correction at the single-domain level. None of the main effects between owning multiple pets and the brain measures were significant. However, we did find a multivariate effect for an interaction between chronological age and owning multiple pets on cortical thickness overall. This interaction appeared to be due to greater effects of owning multiple pets as one gets older. Older pet owners might have more or longer opportunities to interact with pets than younger pet owners, potentially magnifying the effects. Older adults tend to have fewer social roles compared to younger adults and therefore may benefit more from the social, emotional, and physical aspects of pet ownership.

### Pets as protective or selected by the cognitively able?

One criticism of the human-animal interaction literature when using cross-sectional data is the inability to distinguish the direction of the causal relationships having to do with pet ownership. For example, we show here that pet owners (and dog owners, specifically) have better cognitive and brain health than non-pet owners. Our theoretical framework is based on the notion that owning pets leads to repeated and sustained use of specific cognitive processes, thereby strengthening those processes across the adult lifespan. This argument assumes that pet ownership, in part, causes better cognitive and brain health. However, another interpretation is that people who have better cognitive and brain health are more likely to adopt pets. Perhaps only people that have awareness that they can handle caring for a pet choose to do so whereas some people know that the responsibility is too great for them.

The evidence shown here supports both possibilities. On the one hand, most of the associations were not specific to older adults but were also found in younger adults who have not had as long to benefit from sustained pet ownership and might be at their peak cognitive abilities (Hartshorne and Germine, 2015). These main effects might be interpreted as evidence for the “cognitively able select pets” argument. On the other hand, cognitive training tasks and physical exercise have shown that even young adults’ cognition can be improved (Jaeggi et al., 2008; Stroth et al., 2009), suggesting that pet ownership has potential to still serve as a real-world cognitive training scenario. The decelerated brain aging (i.e., lower BrainAGE score) in pet owners might be interpreted as favoring the “enhancement” argument because people do not have direct access to information about their biological age. Interestingly, a study investigating the relationship between one’s subjective age (i.e., how old one feels they are) explained some small variance in BrainAGE (*R^2^* = 0.10; Kwak et al., 2018). The aforementioned interaction between multiple pet ownership and cortical thickness also might be interpreted as favoring the “enhancement” argument because the effects were specific to older adults and not to all age groups that consist of adults with equally high or higher cognitive abilities. Lastly, and perhaps the strongest evidence for enhancement effects, is that many of the specific cognitive and brain effects were significant after controlling for global cognition and global brain health. Thus, while the direction of the causal effects remain limited in this cross-sectional study, we feel the balance of evidence favors an argument that pet ownership enhances cognitive and brain health.

### Limitations

In addition to being unable to fully tease out the causal role that pet ownership has on cognitive and brain health, other limitations are inherent in these analyses. First, we did not have information about attachment to pets or whether the participant was the primary caretaker, both of which might serve as moderating factors that influence dedication and attention to the needs of one’s pet. Second, we did not have information on the age of the pets. Caring for a younger animal versus an older animal would entail very different behaviors and, thus, brain benefits. Lastly, we did not have information about the length of time participants have owned pets. Our theoretical framework assumes that pet ownership would have to be long enough to see sustained changes in cognition and brain health but the time needed to show such effects remain unknown.

### Conclusion and implications

The present study provides strong evidence that pet ownership is associated with better cognitive and brain health with the strongest effects for dog owners. Such evidence is needed to support the rationale and motivation for lifespan interventions to improve brain health before overt decline is present. Currently, nearly every cognitive intervention study using pets or trained animals is aimed at older adults with cognitive decline. If such interventions also show benefits in cognitively normal older adults, it would reveal a type of far-transfer effect to multiple domains of cognition and multiple brain networks, including those involved in processing speed, attention, and episodic memory. It may be that community-based interventions to involve healthy older adults with pets (fostering pets, volunteering at shelters, or pet-assisted exercise such as dog walking) would enhance lifespan cognitive health and meet community-based needs. Notably, while similar life experiences such as raising a child or grandchild appear to have some overlapping qualities as pet ownership, some research suggests that raising children costs rather than aids cognitive health and mortality (Burn and Szoeke, 2015; Bonsang and Skirbekk, 2022). Thus, pet ownership might have multiple unique benefits for adults of all ages.

## Data availability statement

The datasets presented in this article are not readily available because the data collected in this study are owned by The University of Alabama. Interested parties must fill out a data use agreement with UA to access the data or collaborate with the lead author. Requests to access the datasets should be directed to immcdonough@ua.edu.

## Ethics statement

The studies involving human participants were reviewed and approved by the institutional review board at The University of Alabama. The patients/participants provided their written informed consent to participate in this study.

## Author contributions

IM: conceptualization, data curation, formal analysis, investigation, methodology, project administration, visualization, writing–original draft, reviewing, and editing. HE: writing–original draft, reviewing, and editing. NS and RA: conceptualization, writing–reviewing, and editing. All authors contributed to the article and approved the submitted version.

## Funding

This research was funded by The University of Alabama through the College Academy of Research, Scholarship, and Creative Activity to IM and the University of Alabama, Birmingham (#A18-0284-001) to IM. No funds were received for open access publication fees.

## Conflict of interest

The authors declare that the research was conducted in the absence of any commercial or financial relationships that could be construed as a potential conflict of interest.

## Publisher’s note

All claims expressed in this article are solely those of the authors and do not necessarily represent those of their affiliated organizations, or those of the publisher, the editors and the reviewers. Any product that may be evaluated in this article, or claim that may be made by its manufacturer, is not guaranteed or endorsed by the publisher.

## Footnotes

^1^Because perceived SES (measured *via* the MacArthur Scale of Subjective Socioeconomic Status) significantly differed between groups, analyses were re-conducted using this measure as a post-hoc covariate. Perceived SES was not related to the cognitive or brain variables and controlling for it did not alter the results.

^2^BrainAGE analyses often are created using only brain metrics rather than a composite of cognitive and brain metrics. Although brain functioning underlies cognitive performance, a one-to-one mapping of our brain metrics to cognition does not exist. Thus, cognitive performance ultimately reveals additional brain functioning. Nonetheless, re-calculating the BrainAGE scores continued to reveal group differences in pet ownership (*ps*_adj_ < 0.040).
