# Supplementary material for: Pet ownership is associated with greater cognitive and brain health in a cross-sectional sample across the adult lifespan
Source: Front Aging Neurosci. 2022 Oct 20;14:953889. doi: 10.3389/fnagi.2022.953889 (PMC9630635; doi:10.3389/fnagi.2022.953889)

**SUPPLEMENTARY MATERIALS**

Pet Ownership is Associated with Greater Cognitive and Brain Health in a Cross-Sectional Sample across the Adult Lifespan

Ian M. McDonough^1,2^

Hillary B. Erwin^1^

Nancy L. Sin^3^

Rebecca S. Allen^1,2^

^1^The University of Alabama, Department of Psychology

^2^Alabama Research Institute on Aging

^3^University of British Columbia, Department of Psychology

**Supplementary Table 1. Mean Values for Brain Metrics**

| **Brain Network** | **Volume (mm^3)** | **Surface Area (mm^2)** | **Cortical Thickness (mm)** | **RS Connectivity (Z)** |
| --- | --- | --- | --- | --- |
| Dorsal Attention A | 11520.10 | 5270.37 | 2.03 | 3.06 |
| Dorsal Attention B | 8354.73 | 4017.49 | 1.99 | 2.96 |
| Ventral Attention | 11690.62 | 5154.38 | 2.16 | 2.45 |
| Limbic A | 8336.23 | 3457.55 | 2.12 | 3.68 |
| Limbic B | 12332.54 | 3855.72 | 2.61 | 3.73 |
| Default Mode A | 16616.40 | 7338.86 | 2.07 | 2.90 |
| Default Mode B | 17696.95 | 6977.37 | 2.23 | 2.63 |
| Default Mode C | 4159.47 | 1729.45 | 2.24 | 4.18 |
| Default Mode D | 6853.61 | 2713.70 | 2.29 | 4.14 |
| Hippocampus | 3308.82 | - | - | - |

**Supplementary Table 2. Subcortical Brain Regions Associated with each Cortical Network in Resting-State Connectivity Analyses**

| **Network** | **Thalamus** | **Caudate** | **Putamen** | **Pallidum** | **Hippocampus** | **Amygdala** | **Accumbens** |
| --- | --- | --- | --- | --- | --- | --- | --- |
| Dorsal Attention A | 0.798 | 1.086 | 1.799 | 1.079 | 1.706 | 0.467 | 1.004 |
| Dorsal Attention B | 0.812 | 0.694 | 0.468 | 0.837 | 1.136 | 1.044 | 1.056 |
| Ventral Attention | 0.988 | 1.369 | **6.121** | **4.255** | 1.468 | 2.659 | 0.604 |
| Limbic A | 1.125 | **2.278** | 1.886 | 1.500 | 1.187 | 1.605 | **4.292** |
| Limbic B | 0.981 | 1.000 | 2.094 | 1.214 | 5.369 | **8.853** | 0.503 |
| Default Mode A | 1.132 | 2.089 | 1.523 | 1.344 | 0.829 | 1.163 | 2.189 |
| Default Mode B | 1.521 | 2.230 | 1.917 | 1.815 | 1.357 | 0.775 | 0.487 |
| Default Mode C | **2.171** | 1.339 | 1.458 | 1.370 | **7.740** | 3.687 | 1.309 |
| Default Mode D | 0.441 | 1.436 | 0.986 | 0.737 | 1.048 | 1.259 | 2.242 |

**Note.** Bolded values represent highest connectivity of each subcortical brain region out of the tested brain networks.

**Supplementary Figure 1.** Subcortical brain regions most associated with each cortical brain network in resting-state connectivity analyses.


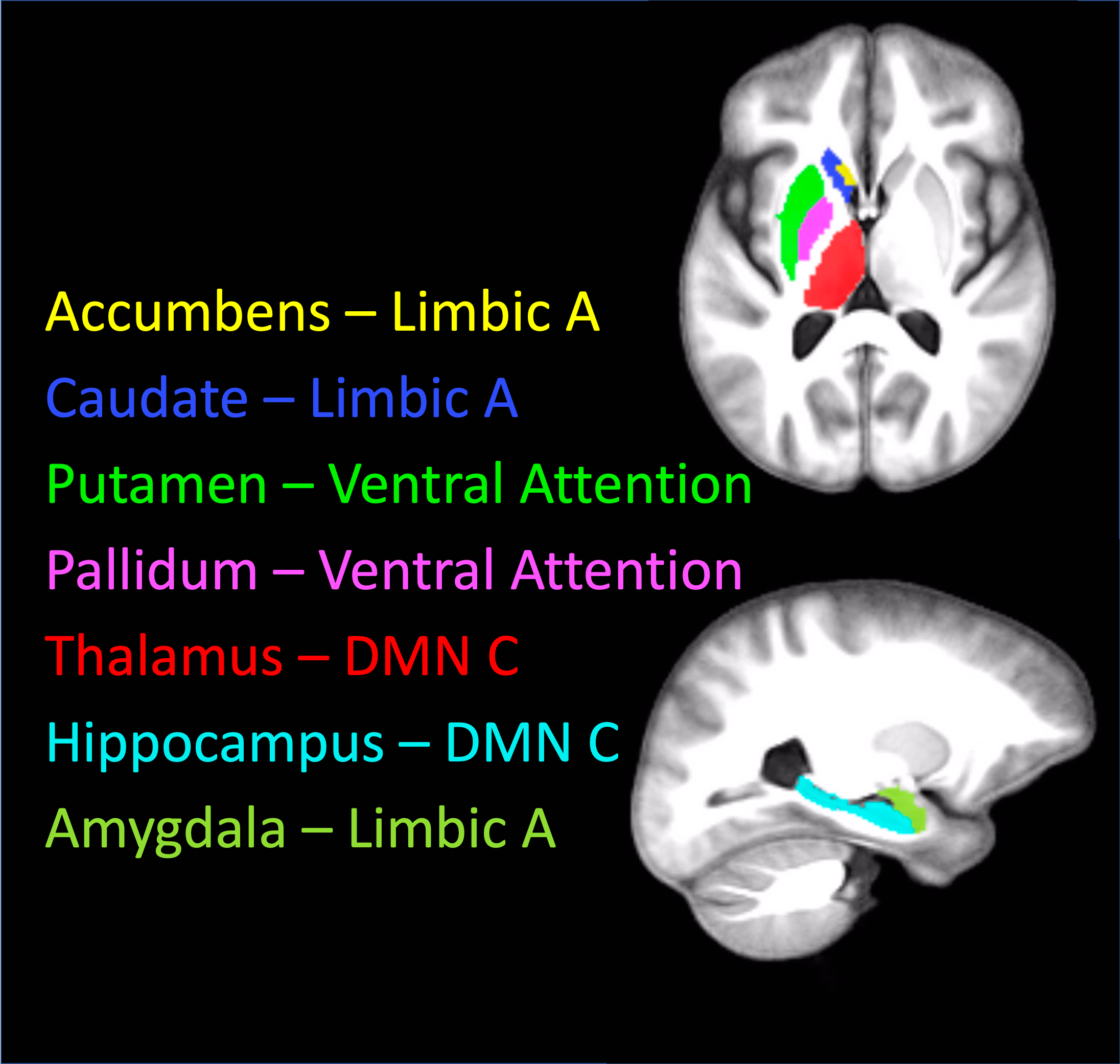

Supplement: Supplementary file 1 [file Data_Sheet_1.docx]
